# Novel rare-earth-free yellow Ca_5_Zn_3.92_In_0.08_(V_0.99_Ta_0.01_O_4_)_6_ phosphors for dazzling white light-emitting diodes

**DOI:** 10.1038/srep10296

**Published:** 2015-05-20

**Authors:** E. Pavitra, G. Seeta Rama Raju, Jin Young Park, Lili Wang, Byung Kee Moon, Jae Su Yu

**Affiliations:** 1 Department of Electronics and Radio Engineering, Kyung Hee University, Yongin 446-701, Republic of Korea; 2 Department of Physics, Pukyong National University, Busan 608-737, Republic of Korea

## Abstract

White light-emitting diode (WLED) products currently available on the market are based on the blue LED combined with yellow phosphor approach. However, these WLEDs are still insufficient for general illumination and flat panel display (FPD) applications because of their low color-rendering index (CRI < 75) and high correlated color temperature (CCT = 6000 K). Although near-ultraviolet (UV) LED chips provide more efficient excitation than blue chips, YAG:Ce^3+^ phosphors have very weak excitation in the near-UV spectral region. Hence, there is an increasing demand for novel yellow phosphor materials with excitation in the near-UV region. In this work, we report novel self-activated yellow Ca_5_Zn_3.92_In_0.08_(V_0.99_Ta_0.01_O_4_)_6_ (CZIVT) phosphors that efficiently convert near-UV excitation light into yellow luminescence. The crystal structure and lattice parameters of these CZIVT phosphors are elucidated through Rietveld refinement. Through doping with In^3+^ and Ta^5+^ ions, the emission intensity is enhanced in the red region, and the Stokes shift is controlled to obtain good color rendition. When a near-UV LED chip is coated with a combination of CZIVT and commercial blue Ba_0.9_Eu_0.1_MgAl_10_O_17_ phosphors, a pleasant WLED with a high CRI of 82.51 and a low CCT of 5231 K, which are essential for indoor illumination and FPDs, is achieved.

In recent years, light-emitting diodes (LEDs) have been developed across the ultraviolet (UV), visible, and infrared wavelengths, and their use has been extended from interior and exterior lighting to display applications^1^. Compared with incandescent and fluorescent light sources, LED-based white-light emitters have significant potential for general illumination applications because of their advantages such as high efficiency, long life span, high reliability, reduced power consumption, cost effectiveness, and environmental benignity due to less thermal radiation and no mercury^2^,^3^,^4^,^5^,^6^,^7^. To date, the majority of white LEDs (WLEDs) currently available on the market are blue-emitting GaN LEDs (450–460 nm) coated with yellow-emitting Y_3_Al_5_O_12_:Ce^3+^ (YAG:Ce^3+^) phosphor; however, these YAG-based WLEDs suffer from a poor color-rendering index, low color reproducibility, and thermal quenching at elevated temperatures^8^,^9^,^10^,^11^,^12^. Recently, WLEDs fabricated using near-UV LED chips have become a new topic of considerable research interest because they provide a higher excitation efficiency (similar to that of a fluorescent light tube) than blue chips^8^,^13^,^14^,^15^,^16^. The electroluminescence (EL) intensity of the blue band increases considerably more rapidly than that of the longer wavelength band, and thus, the output power of blue LED tends to saturate at high driving currents^17^. Therefore, the color temperature easily changes, and the color-rendering index is also low due to the spectral non-uniformity^18^. However, it is not possible to fabricate white-LEDs by coating YAG:Ce^3+^ phosphors on near-UV LED chips because the photoluminescence (PL) excitation (PLE) intensity of the YAG:Ce^3+^ phosphor is very weak in the deep blue or near-UV (350–420 nm) spectral region^19^. Thus, the demand for novel phosphors with an excitation band in the near-UV spectral region has been rapidly increasing^20^.

At present, alkaline earth vanadates have been the focus of significant attention due to their enhanced chromaticity in solid-state lighting devices^21^. The vanadate cluster [VO_4_]^3−^, in which the central metal ion is coordinated by four oxygen ions in tetrahedral (T_d_) symmetry, serves as an efficient luminescent center^22^. Interestingly, it has been reported that some of these vanadates have unusual luminescent properties, such as broad and intense oxygen-to-metal charge transfer (CT) bands typically observed in the near-UV region, thereby allowing for the efficient capture of emissions over a large wavelength range (400–700 nm). Moreover, this type of self-activated vanadate phosphor possesses many advantages over rare-earth-activated phosphors. Compared with conventional phosphors, self-activated vanadate phosphors are economical and energy-saving compounds because of their relatively low annealing temperatures of 600 to 800 °C. Additionally, these compounds have a broadband emission spectrum that covers the entire visible region to obtain a high color-rendering index (CRI)^23^. Therefore, a self-activated vanadate with a proper composition is essential for the fabrication of white-light phosphors.

In this work, we report a novel Ca_5_Zn_4(1-x)_In_4x_(V_1-y_Ta_y_O_4_)_6_ phosphor, which provides a yellow emission upon near-UV excitation. This phosphor is perfectly suitable for fabricating near-UV based WLEDs when combined with a blue phosphor. In contrast to conventional rare-earth schemes, non-rare-earth elements, namely, In^3+^ and Ta^5+^ ions, are used as dopants. This is the first report on the Ca_5_Zn_4(1-x)_In_4x_(V_1-y_Ta_y_O_4_)_6_ yellow phosphor. This phosphor exhibits an internal quantum efficiency that is 86% higher than that of its parent host lattice, i.e., Ca_5_Zn_4_(VO_4_)_6_ (CZV), under similar synthetic conditions, and that is 156% higher than that in an earlier report^24^, which establishes its high potential for solid-state lighting applications. The structural properties were investigated by performing Rietveld refinements on X-ray diffraction (XRD) patterns. The optical properties of these self-activated vanadate phosphors were examined in detail through PLE and PL emission measurements. A WLED of desirable quality was fabricated by taking the appropriate amounts of the novel yellow Ca_5_Zn_3.92_In_0.08_(V_0.99_Ta_0.01_O_4_)_6_ (CZIVT) and commercial blue Ba_0.9_Eu_0.1_MgAl_10_O_17_ (BAM) phosphors in combination with a near-UV LED chip. The fabricated WLED exhibited unique properties, such as a high CRI (82.51) and a low correlated color temperature (5231 K), which are prerequisites for general illumination and flat panel display applications.

## Results and Discussion

**Figure 1:**
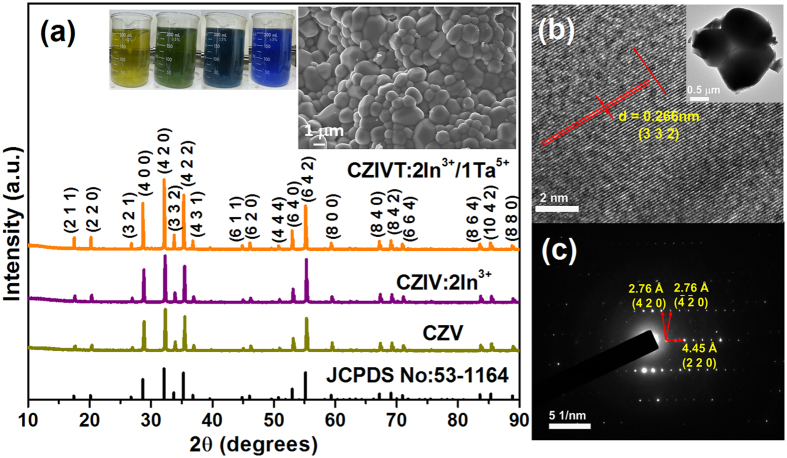
XRD patterns and SEM and TEM images of CZV, CZIV, and CZIVT phosphors. **** (**a**) XRD patterns of the CZV, CZIV, and CZIVT phosphors (insets show the changes in solution color during the heating process and the SEM image). (**b**) HRTEM images and (**c**) SAED patterns of the CZIVT phosphors (inset shows the TEM image of the corresponding particles)

The morphological properties of the CZIVT self-activated phosphors annealed at 800 °C were examined using field-emission scanning electron microscope (FE-SEM) and transmission electron microscope (FE-TEM) images. As indicated by the SEM image shown in the inset of Fig. 1(a), the particles appeared to be crowded with an almost spherical morphology, which is clearly helpful for preventing the scattering of light. Consequently, more proficient output light, which is required for solid-state lighting applications, was obtained. Figure 1(b,c) presents the high-resolution TEM (HRTEM) image and selected area electron diffraction (SAED) pattern of individual particles (shown in the inset of Fig. 1(b)). The HRTEM image and the corresponding SAED pattern confirmed the single-crystalline nature of the CZIVT phosphor without any significant defects or secondary phases. The d-spacings are approximately 2.76 and 4.45 Å, corresponding to the (4 2 0) and (2 2 0) principal planes.

Figure 1(a) shows the XRD patterns of the CZV, Ca_5_Zn_3.92_In_0.08_(VO_4_)_6_ (CZIV), and CZIVT phosphors annealed at 800 °C. All of the diffraction peaks were well indexed to the cubic garnet structure according to the standard JCPDS card no. 53-1164 and space group Ia-3d (230). First, we recorded XRD patterns of CZV phosphors doped with different concentrations of In^3+^ and Ta^5+^ ions. The XRD patterns of CZIV phosphors with different concentrations of In^3+^ ions were measured, as shown in the supplementary information (Fig. S1). From the measured diffraction pattern, no impurity peaks appeared when up to 2 mol% In^3+^ ions was doped into the Zn site. However, with further increases in the In^3+^ ion concentration, an impurity peak appeared at approximately 30°, and this peak was identified as In_2_O_3_ according to JCPDS card no. 71-2194. This impurity peak appeared due to exceeding the solubility limit of In^3+^ to Zn^2+^ ions caused by their different oxidation states. Therefore, 2 mol% was chosen as the optimum concentration of In^3+^ ions for Zn^2+^ ion sites. To obtain the redshift and richness in excitation and emission process, different concentrations of Ta^5+^ ions were doped into the V^5+^ ion sites because both TaO_4_ and VO_4_ have similar chemical properties, with the exception of their coordination environment. The resulting XRD patterns are shown in the supplementary information (Fig. S2). However, at a Ta^5+^ ion concentration above 1 mol%, some impurity peaks were observed, which were identified as Ta_2_O_5_ peaks based on JCPDS card no. 73-2323. This impurity may be caused by the crystal field disturbance resulting from the different coordination environments of V^5+^ (four-fold) and Ta^5+^ (six-fold) ions and from the smaller ionic radius of V^5+^ (0.355 Å) ions compared to that of Ta^5+^ (0.64 Å). Thus, without affecting the structural properties of CZV, CZIVT was developed as a new phosphor material for solid-state lighting applications. Further details regarding the crystal structure and effects of In^3+^ and Ta^5+^ ions are presented in the following sections.

**Figure 2:**
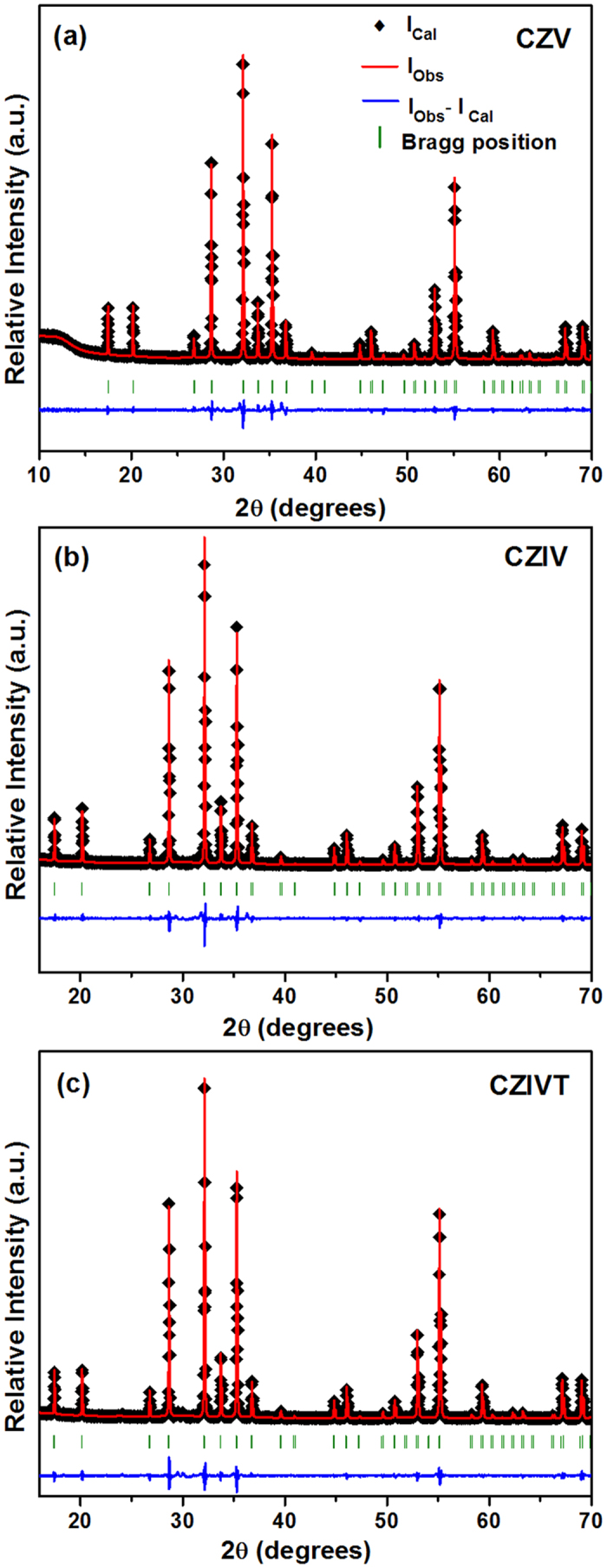
Rietveld refinement . (**a**) CZV, (**b**) CZIV, and (**c**) CZIVT phosphors.

**Figure 3:**
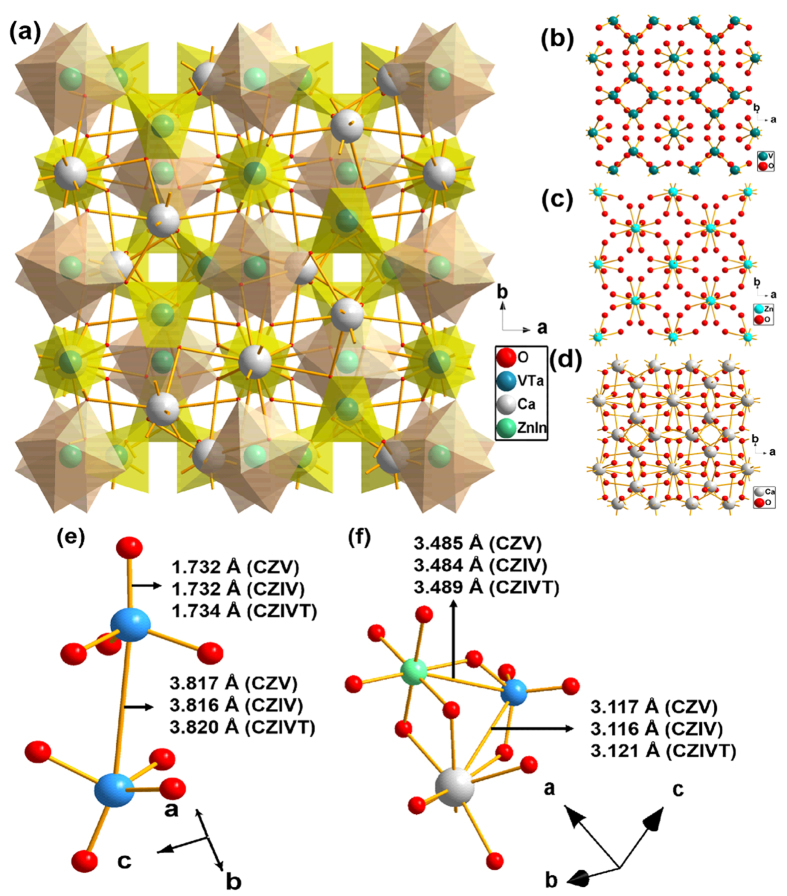
Electronic structure illustrations. **** (**a**) CZIVT unit cell. The unit cell structures for individual bonds of V-O, Zn-O, and Ca-O are shown in (**b**), (**c**), and (**d**), respectively. Comparisons of the V-O, V-V, V-Zn, and V-Ca bond lengths for CZV, CZIV, and CZIVT phosphors are shown in (**e**) and (**f**).

To confirm that the self-activated vanadate phosphors formed in the cubic phase, Rietveld refinement was performed on the XRD patterns using the software General Structure Analysis System (GSAS). Figure 2(a–c) presents the Rietveld refinement results for the CZV, CZIV, and CZIVT phosphors, respectively, and their crystallographic data are summarized in Tables 1 and 2. The observed and calculated results were similar to those reported in the literature under JCPDS card no. 53-1164, in which the CZV phosphor adopted a cubic structure with space group Ia-3d (230). The obtained unit cell parameters were as follows: a = 12.4668 Å, V = 1937.60 Å^3^ for CZV; a = 12.4636 Å, V = 1936.13 Å^3^ for CZIV; and a = 12.4784 Å, V = 1943.02 Å^3^ for CZIVT. Using Diamond software, the ideal unit cell was modeled using the acquired atomic coordinates, as shown in Fig. 3(a). In this vanadate garnet structure, Zn^2+^ and Ca^2+^ ions occupy the six-fold octahedral (Fig. 3(c)) and eight-fold dodecahedral (Fig. 3(d)) sites, respectively, whereas the V^5+^ ions occupy the four-fold tetrahedral site (Fig. 3(b))^24^,^25^,^26^. Without any disturbance in phase formation, 2 mol% In^3+^ ions and 1 mol% Ta^5+^ ions are also substituted in the Zn^2+^ and V^5+^ sites, respectively. Note that the structure of the [VO_4_]^3−^ tetrahedron is distorted to some extent compared to the ideal tetrahedron. This distortion depends on the deviation in the four V-O bond lengths and six O-V-O bond angles in the [VO_4_]^3−^ tetrahedron^27^,^28^. In contrast, the [VO_4_]^3−^ tetrahedra are isolated from each other in the crystal structure. According to the literature, a shorter V-O or V-V distance results in a larger distortion of the [VO_4_]^3−^ tetrahedron from the tetrahedral symmetry^24^. In this work, the doping of Ta^5+^ ions into the tetrahedral site somewhat increases the V-O and V-V bond lengths compared to those in the CZV and CZIV phosphors. However, the increases in bond length between V and metal ions are relatively larger than those of the V-O and V-V bond lengths. To provide a better understanding, the deviations in the bond lengths are shown in Fig. 3(e,f), respectively. The results indicate that the Ta^5+^ ions exhibit an effect on the crystallographic environment and that these modifications provide desirable optical properties. The bond length variations arising from doping In^3+^ and Ta^5+^ ions into the CZV host lattice of each phosphor are shown in Table 2.

According to the obtained crystallographic data, the volume of the unit cell was altered by doping with In^3+^ and Ta^5+^ ions due to their different ionic radii and coordination environments^29^. The total ionic radius of the metal ions in a unit cell was 10.69 Å for the pure CZV host material. By doping In^3+^ ions in the Zn^2+^ sites (CZIV), the total ionic radius of the metal ions was found to be 10.6948 Å, which is similar to that of the host material due to the nearly equal ionic radii of the In^3+^ (0.8 Å) and Zn^2+^ (0.74 Å) ions. Likewise, in the case of Ta^5+^ ion-doped CZIV phosphors (i.e., CZIVT), the obtained total ionic radius of the metal ions was increased to 10.7119 Å due to the larger ionic radius of Ta^5+^ ions (0.64 Å) than V^5+^ ions (0.355 Å). Thus, by doping Ta^5+^ ions into the V^5+^ sites, a lattice expansion occurred such that the obtained unit cell parameters, volume, and bond lengths were enlarged for the CZIVT phosphor.

Initially, the PLE and PL emission spectra of the CZV, CZIV, and CZIVT phosphors with different concentrations of dopant (In^3+^ and Ta^5+^) ions were measured by monitoring the corresponding emission and excitation wavelengths, respectively, as shown in the supplementary information (Fig. S3). The optimal dopant concentration was found to be 2 mol% for In^3+^ ions and 1 mol% for Ta^5+^ ions. Above the optimal concentrations of In^3+^ and Ta^5+^ ions, the excitation emission intensities gradually decrease. This decrease might be attributed to the concentration quenching and/or development of impurity peaks, as mentioned in the XRD section. Therefore, the CZIVT phosphors doped with optimal concentrations of In^3+^ and Ta^5+^ ions were employed for further characterizations.

**Figure 4:**
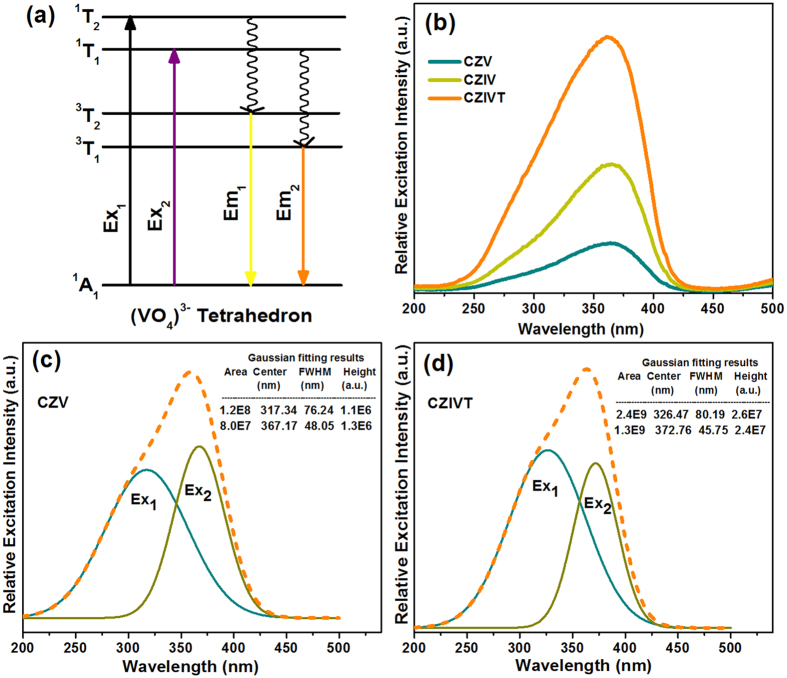
Energy transfer and excitation comparisons of CZV, CZIV and CZIVT phosphors. **** (**a**) Schematic diagram of the possible energy transitions that occurred in the (VO_4_)^3−^ tetrahedron. (**b**) PLE spectra of CZV host, CZIV, and CZIVT phosphors obtained by monitoring the corresponding emission wavelengths at 538, 540, and 546 nm, respectively. The Gaussian fits to the spectra of the CZV host and CZIVT phosphors are shown in (**c**) and (**d**).

Typically, the ground-state molecular orbital of V^5+^ ions with Td symmetry is characterized as the ^1^A_1_ state, which is completely symmetric due to the vacant d orbital (d^0^), and the excited states are characterized as ^1^T_1_, ^1^T_2_, ^3^T_1_, and ^3^T_2_ levels. A schematic illustration of the possible excitation and emission transitions is presented in Fig. 4(a). The excitation spectrum consists of two absorption bands caused by the partly allowed spin-forbidden transitions from the ground state ^1^A_1_ to the excited states ^1^T_2_ and ^1^T_1_, respectively. Regarding the two absorption bands in the excitation spectrum, the minor intensity band, which occurs predominantly in the spectrum corresponding to the ^1^A_1_ → ^1^T_2_ (Ex_1_) transition, is also known as a shoulder peak, and the major intensity band is due to the ^1^A_1_ → ^1^T_1_ (Ex_2_) transition. The positions and intensities of the two bands in the excitation spectrum may depend on two factors: the first is the concentration of the VO_4_^3−^ groups, and the second is the nature of the host lattice^30^.

Figure 4(b) compares the intensities of the PLE spectra of the CZV, CZIV, and CZIVT phosphors monitored at the corresponding emission wavelengths. Here, the CZV, CZIV, and CZIVT phosphors exhibited broadband excitation spectra ranging from 250 to 420 nm with band maxima at 358, 363, and 369 nm, respectively. The CZV excitation band contains two absorption peaks at 317 and 367 nm, which correspond to Ex_1_ and Ex_2_, respectively. The corresponding Gaussian components are shown in Fig. 4(c). By doping with the optimized concentrations of In^3+^ and Ta^5+^ ions, we are able to progressively shift the position of the absorption bands from 317 to 326 nm for Ex_1_ and from 367 to 373 nm for Ex_2_. However, this redshift changes the magnitude of the Stokes shift because, according to the literature, the V-O bond distance plays a key role in the energy of [VO_4_]^3−^ CT transitions. Therefore, the redshift might occur due to the increased V-O bond distance (1.736 Å) for CZIVT phosphors compared to the V-O bond distance (1.732 Å) in CZV phosphors. To provide a better understanding, the Gaussian fits of the bands of CZIVT phosphors are shown in Fig. 4(d). Moreover, the intensity of the predominant absorption band (Ex_1_) of the CZIVT phosphors was increased compared to the absorption band of pure CZV. The corresponding full width at half maximum (FWHM) values were 76.24 nm for the CZV host lattice and 80.19 nm for the CZIVT phosphors. In addition, the Ta^5+^ ions present an excitation band at approximately 280 nm. Thus, by doping with Ta^5+^ ions, the intensity of the absorption band corresponding to Ex_1_ was increased. Consequently, by doping with transition metal ions (In^3+^ and Ta^5+^), the intensity of the total excitation spectrum was increased, and the position of the band was also shifted to the longer wavelength region. This excitation wavelength range is consistent with the emission of near-UV LED chips (360–420 nm). Moreover, the CZIVT excitation spectrum clearly differed from previously reported phosphors based on near-UV excitation because CZIVT has no absorption band in the blue region. The intensity of the excitation band began to rapidly decrease at wavelengths above 385 nm and reached its original state at 411 nm. A comparison of the thermal stabilities between CZV (ΔS = 9.2 cm^−1^) and Ca_5_Mg_4_(VO_4_)_6_, also called CMV, (ΔS = 8.5 cm^−1^) phosphors has been reported^24^, in which it was concluded that the decreased ΔS increases the thermal stability. The ΔS for the CZIVT phosphor was estimated to be 7.65 × 10^3^ cm^−1^, which is considerably smaller than that previously reported for CZV and CMV phosphors. However, Daicho *et al.*^31^ reported that phosphors with larger ΔS values are better for white-light emission with a uniform hue because these phosphors show absorption between the UV and NUV regions. The ΔS values for (Ca_0.37_Sr_0.53_Eu_0.10_)_7_(SiO_3_)_6_Cl_2_, (Ba,Sr)_2_SiO_4_:Eu^2+^, and (Sr,Ca)AlSiN_3_:Eu^2+^ were 6.9 × 10^3^, 3.2 × 10^3^, and 2.6 × 10^3^ cm^−1^, respectively, which are smaller than that of the present CZIVT phosphors. Therefore, the CZIVT phosphors are capable of converting near-UV emission into white light when mixed with an appropriate amount of blue phosphor, which is essential for efficient WLEDs with a high CRI and desirable CCT values.

**Figure 5:**
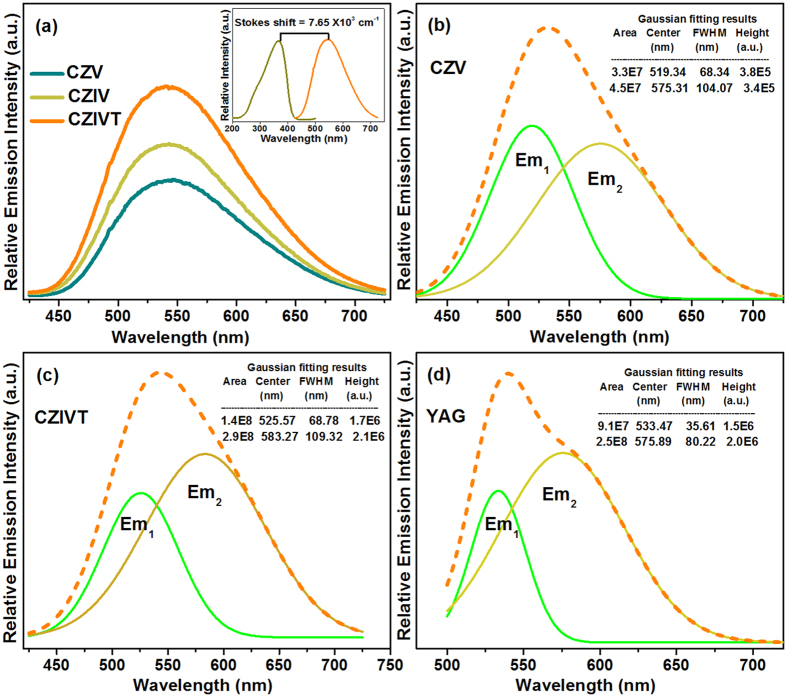
Luminescence properties of CZV, CZIV, CZIVT, and YAG: Ce^3+^ phosphors. (**a**) PL emission spectra of CZV host, CZIV, and CZIVT phosphors obtained by monitoring the excitation wavelength at 363 nm. (Inset shows the Stokes shift for CZIVT phosphors). The Gaussian fits to the spectra of CZV, CZIVT, and YAG: Ce^3+^ phosphors are shown in (**b**), (**c**), and (**d**), respectively.

The PL emission spectra at different excitation wavelengths are presented in the supplementary information (Fig. S4). It was observed that the emission intensities were identical, except for the CZV excitation (358 nm), suggesting that these phosphors are better suited for near-UV chip (360–385 nm)-based WLEDs. Figure 5(a) presents the PL emission spectra of the CZV, CZIV, and CZIVT phosphors under excitation at 363 nm. From the PL emission spectra, it is clear that all three phosphors exhibited the characteristic broadband emission spectra in the wavelength range from 420 to 720 nm with band maxima at 538 nm (CZV), 540 nm (CZIV), and 546 nm (CZIVT). This type of broadband emission of vanadate phosphors is attributed to the CT of an electron from the 2*p* orbital of the oxygen atom to the vacant 3*d* orbital of V^5+^ ions in the tetrahedral [VO4]^3−^ group^22^,^32^. It is well known that, for the vanadate phosphors, the emission band contains two broad band peaks corresponding to the ^3^T_2_ → ^1^A_1_ and ^3^T_1_ → ^1^A_1_ transitions of the [VO_4_]^3−^ group. The emission bands from these two transitions are overlapped, and they appear to be a single band because of the very small (approximately 0.06 eV) energy difference between the ^3^T_1_ and ^3^T_2_ excited states. Therefore, it is very difficult to distinguish between these two bands in the emission spectrum with the naked eye^30^. To observe the difference between the two (^3^T_2_ → ^1^A_1_ and ^3^T_1_ → ^1^A_1_) transitions, the emission spectrum of the CZV phosphor was first deconvolved into two bands using Gaussian fittings, as shown in Fig. 5(b). The first emission band with a band maximum at 519 nm is attributed to the electronic transition ^3^T_2_ → ^1^A_1_ (Em_1_), and the second emission band with a band maximum at 575 nm corresponds to the ^3^T_1_ → ^1^A_1_ (Em_2_) transition of the [VO_4_]^3−^ tetrahedrons. Moreover, the Em_1_ band that occurred at 519 nm was more intense than that of the Em_2_ band at 575 nm, and the corresponding FWHM values were found to be 68 and 104 nm, respectively. The emission spectrum of the CZIVT phosphor was also deconvolved into two bands, and the resulting Gaussian components are presented in Fig. 5(c). The peak positions of both emission bands (Em_1_ and Em_2_) exhibited a significant redshift compared to the CZV phosphor. The resulting band maxima were found to occur at 525 and 583 nm with FWHM values of 68 and 109 nm, respectively, for the Em_1_ and Em_2_ bands. This result was in good agreement with the results of Ronde *et al.*^30^ and Huang *et al.*^24^ These authors reported that the energy of the [VO_4_]^3−^ CT transition depends on the V-O bond length. A decrease in transition energies, ΔE(t_1_-2e), occurs if the V-O bond length increases, which provides the redshift for both the Em_1_ and Em_2_ emission positions. Here, by doping with Ta^5+^ ions, the V-O bond length increases, but this increase is less compared to the V-metal ion bond lengths. Moreover, the intensity and broadness of the emission band centered at 583 nm (Em_2_) were significantly increased compared to the CZV phosphor, which might be attributed to the existence of Ta^5+^ ion emission at approximately 584 nm^33^. Thus, the doping of Ta^5+^ ions into the V^5+^ sites causes an enhancement of the emission spectrum with extended broadness in the red region. Additionally, the doping of In^3+^ ions into the Zn^2+^ sites enhances the blue emission intensity^34^. Consequently, by doping with transition metal ions (In^3+^ and Ta^5+^), the intensity and broadness of the emission spectrum of CZIVT phosphors are improved.

To explore the CZIVT phosphor richness, the emission spectrum of a commercial YAG:Ce^3+^ phosphor was measured under similar conditions. The PL emission spectrum of the YAG:Ce^3+^ with Gaussian fitting curves was compared with that of the CZIVT phosphor, as shown in Fig. 5(d). By comparing the emission spectra of the YAG:Ce^3+^ phosphor and the CZIVT phosphor, the FWHM values of the Em_1_ and Em_2_ bands were found to be 36 and 80 nm, respectively, for the YAG:Ce^3+^ phosphor and 68 and 109 nm, respectively, for the CZIVT phosphor. This result indicates that the YAG:Ce^3+^ phosphor exhibits smaller emission regions. Due to the overlapped excitation and emission of YAG:Ce^3+^ and the small ΔS, a non-uniform hue was produced. However, due to the large ΔS without overlapping of the emission and excitation bands with enhanced intensity and a broadened red region, the CZIVT phosphors are expected to provide a uniform hue with a high color-rendering index (CRI) and a good correlated color temperature (CCT).

**Figure 6:**
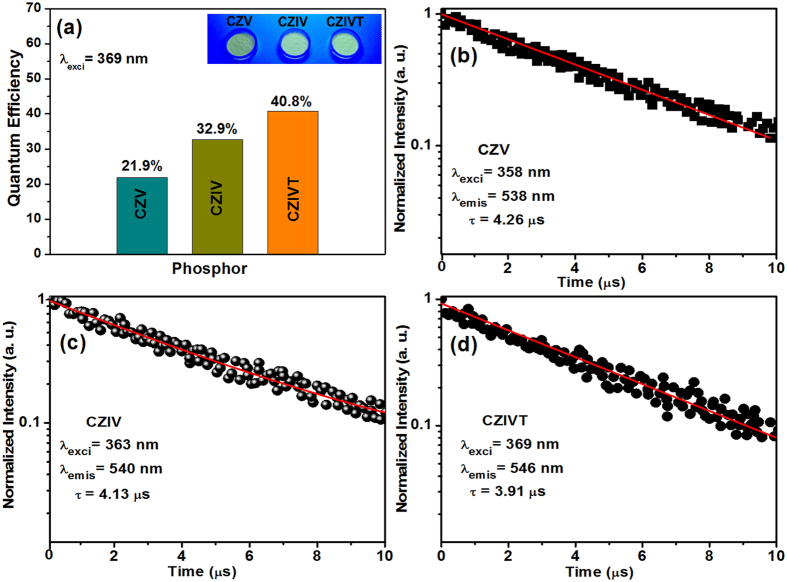
Quantum efficiencies and decay times of CZV, CZIV, and CZIVT. **** (**a**) Quantum efficiencies of the CZV host, CZIV, and CZIVT phosphors under 369 nm excitation. The decay curves of the CZV host, CZIV, and CZIVT phosphors as a function of their corresponding excitation and emission wavelengths are shown in (**b**), (**c**) and (**d**) respectively.

Quantum efficiency is one of the most important properties of phosphors for their applications in various fields. The attained quantum efficiency values were 21.9, 32.9, and 40.8% for the CZV, CZIV, and CZIVT phosphors, respectively, while exciting with 369 nm (for comparison purposes) at room temperature, as shown in Fig. 6(a). From these results, it is observed that the quantum efficiency was enhanced as a result of doping with transition metal ions. Nakajima *et al.* reported that the quantum efficiency of vanadate was strongly enhanced by the isolated (VO_4_)^3−^ tetrahedrons in the lattice with a short V-V bond length^28^. As mentioned above, doping with In^3+^ and Ta^5+^ ions has a considerable effect on the bond length. Overall, the increase of the V-V and V-O bond lengths is relatively smaller than that of the bond lengths between the V and metal ions, indicating that the V-V and V-O interactions are larger than that of the V-metal ions. This explanation is consistent with 24 and 27]. However, the obtained quantum efficiency was considerably higher than previously reported results and also extremely higher than that of sulfide-based phosphors. The decay curves of the CZV, CZIV, and CZIVT phosphors recorded by monitoring the excitation wavelengths at 358, 363, and 369 nm and the emission wavelengths at 538, 540, and 546 nm are shown in Fig. 6(b–d). The decay curves were well fitted to a single exponential function, and the obtained lifetimes were approximately 4.26, 4.13, and 3.91 μs for the CZV, CZIV, and CZIVT phosphors, respectively. Thus, the short decay time makes the CZIVT a very attractive yellow-emitting phosphor for WLEDs.

**Figure 7:**
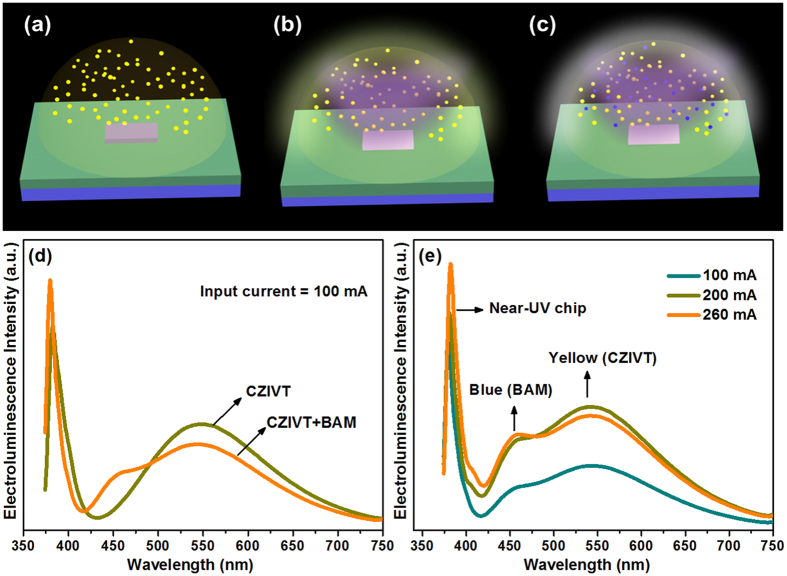
Schematic illustrations and EL spectra of CZIVT + BAM. **** (**a**) Schematic diagram of a near-UV LED chip coated with CZIVT before applying the current. The yellow-green and white-light emission from the pc-LEDs after applying the input current are shown in (**b**) and (**c**), (**d**) EL spectra of CZIVT and the mixture of CZIVT + BAM phosphors-coated near-UV LED chip under a 100 mA input forward-bias current. (**e**) EL spectra of the fabricated WLED at different input forward-bias currents from 100 to 260 mA.

To estimate the practical applicability of yellow CZIVT phosphors, we fabricated two phosphor-converted (pc) LEDs. A schematic diagram of the LED package is shown in Fig. 7. Sample 1 was prepared as a near-UV LED chip coated with a yellow (CZIVT) phosphor, and sample 2 was a near-UV LED chip coated with a mixture of yellow CZIVT+ commercial blue BAM phosphors (commercial product KX661, from Kasei Optonix Ltd). For both samples, the phosphor powders were homogeneously distributed in a silicone encapsulant. The proportions of CZIVT and BAM phosphors play an important role in obtaining efficient WLEDs. A schematic diagram of the LED package before applying the input forward-bias current is shown in Fig. 7(a). After applying an input forward-bias current of approximately 100 mA, a yellow-green emission and a pleasant white emission were observed from samples 1 and sample 2, respectively, as shown in Fig. 7(b,c).

The EL spectra of the two pc-LEDs under an input-forward bias current of approximately 100 mA are shown in Fig. 7(d). Both EL spectra contain two distinct emission bands, where the short-wavelength EL peak located at 380 nm originated directly from the near-UV chip and the broad long-wavelength EL band with a band maximum at 547 nm was emitted by the CZIVT phosphor or combination of CZIVT+ BAM phosphors followed by the absorption of near-UV light. This band position is in good agreement with the normal PL spectra. To further investigate the potential of the fabricated WLED (sample 2), the characteristics of sample 2 were observed under different input forward-bias currents from 100 to 260 mA; the corresponding EL spectra are presented in Fig. 7(e). In the EL spectra, the emission band intensity increased as the input forward-bias current increased from 100 to 200 mA. There was no observed shift in the EL peak positions of three visible emission bands.

As the input forward-bias current was further increased to 260 mA, the intensities of the blue and yellow emission bands appeared to be nearly equal. This property is very useful for obtaining glare-free and pleasant white-light emission. At a forward-bias current of 260 mA with an operating voltage of 4 V, the luminous flux of the white light-emitting CZIVT + BAM was found to be approximately 48 lm. We can also determine the CRI and CCT from these spectra, and the obtained CRI and CCT values are presented in Table 3.

In general, the CRI is independent of the CCT. However, for sample 2, as the input forward-bias current increased from 100 to 260 mA, the CRI values and the CCT values increased. When the input forward-bias current was 100 mA, the CRI and CCT values were 70.70 and 4757 K, respectively, for sample 1 and 78.10 and 4950 K, respectively, for sample 2. As the input forward-bias current increased from 100 to 260 mA, the CRI value increased from 78.10 to 82.51 and the CCT value increased from 4950 K to 5231 K for sample 2. It is well known that the commercially available WLEDs fabricated by combining the YAG:Ce^3+^ phosphor with a blue InGaN chip suffer from a low CRI of ≤75 and a high CCT of 7756 K. Compared with these commercial WLEDs, our WLED exhibited a higher CRI of 82.51 and a lower CCT of 5231 K, which are necessary parameters for general illumination and optical display applications.

**Figure 8:**
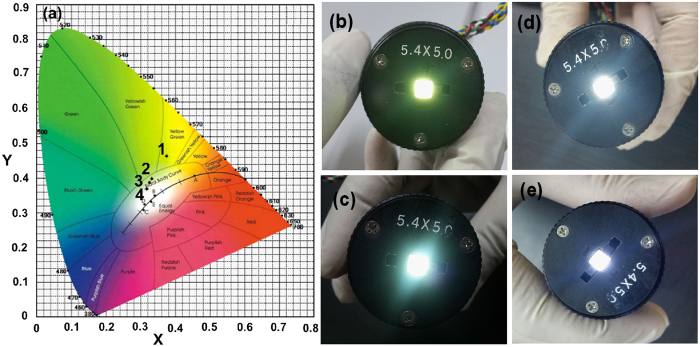
CIE chromaticity coordinates and fabricated LEDs. **** (**a**) CIE coordinates for sample 1 and sample 2 [(1) sample 1 at 100 mA (0.37, 0.46), (2) sample 2 at 100 mA (0.33, 0.39) (3) sample 2 at 200 mA (0.32, 0.38), and (4) sample 2 at 260 mA (0.31, 0.37)]. (**b**) Yellow-green emission from sample 1 under a 100 mA input current. The variation in the white emission from sample 2 under different input currents of 100, 200, and 260 mA are shown in (**c**), (**d**), and (**e**), respectively.

The Commission International De I’Eclairage (CIE) chromaticity coordinates of sample 1 and sample 2 are shown in Fig. 8(a). Sample 1 showed chromaticity coordinates in the yellow-green region with x = 0.3749 and y = 0.4644 under an input forward-bias current of 100 mA (as shown in Fig. 8(b)). Sample 2 exhibited chromaticity coordinates in the white region with x = 0.3325 and y = 0.3989 at 100 mA, x = 0.3259 and y = 0.3877 at 200 mA, and x = 0.3174 and y = 0.3700 at 260 mA of input forward-bias current. The white-light emissions from the corresponding LEDs are shown in Fig. 8(c–e). However, as the input forward-bias current increased, the CIE chromaticity coordinates shifted to the day-light region, exhibiting high-quality white emission with higher CRI values. The variation in the white emission is clearly observed in Fig. 8(e), and a pleasant white emission was achieved.

## Conclusion

We have successfully obtained novel self-activated yellow CZIVT phosphors for near-UV-based WLEDs. The Rietveld refinement confirmed that these phosphors possessed a pure cubic garnet structure, and the HRTEM and SAED patterns indicated that the particles were single crystalline. The quantum efficiency was improved and considerably higher than that of the parent host, i.e., CZV phosphor, and the emission band intensity was increased in the red region by doping with In^3+^ and Ta^5+^ ions. The excitation wavelength range of the CZIVT phosphors is well matched with the emission of near-UV LED chips, which is suitable for converting near-UV emission into white light. The CZIVT phosphors exhibited a yellow-green emission with a CRI = 70.70 and a CCT = 4757 K by combining with a near-UV LED chip. A WLED of desirable quality with a high CRI of 82.51 and a low CCT of 5231 K, with a reasonable luminous flux of 48 lm, was fabricated by combining the CZIVT phosphor with a commercial blue phosphor. This novel CZIVT phosphor is expected to be a promising candidate for the realization of solid-state lighting.

## Methods

### Synthesis of yellow phosphor

Ca_5_Zn_4(1-x)_In_4x_(V_1-y_Ta_y_O_4_)_6_ phosphors were prepared through a sol-gel method using stoichiometric amounts of calcium nitrate tetrahydrate [Ca(NO_3_)_2_·4H_2_O] (Sigma-Aldrich, ≥99.0%), zinc nitrate hexahydrate [Zn(NO_3_)_2_·6H_2_O] (Sigma-Aldrich, 98%), ammonium metavanadate [NH_4_VO_3_] (Sigma-Aldrich, 99.96%), indium nitrate hydrate [In(NO_3_)_3_·xH_2_O] (Sigma-Aldrich, 99.99%), tantalum chloride [TaCl_5_] (Sigma-Aldrich, 99.8%), and citric acid [HOC(COOH)(CH_2_COOH)_2_] (Sigma-Aldrich, ≥99.5%). The concentration ratio of citric acid and metal ions used in this experiment was 1:2. Initially, the parent host lattice Ca_5_Zn_4_(VO_4_)_6_ (CZV) was synthesized by dissolving 5 mmol of calcium nitrate, 4 mmol of zinc nitrate, 6 mmol of ammonium metavanadate, and 30 mmol citric acid in 200 ml of de-ionized (DI) water, which was stirred for 30 min using a magnetic stirrer. Then, this solution was heated on a hot plate with continuous magnetic stirring, and the solution temperature was maintained at 80 °C. The beaker was initially closed with a cap, and after 30 min of heat treatment, the color of the solution turned into green and then blue, as shown in the inset of Fig. 1(a). Then, the cap was removed, and the heat treatment was continued until a bluish wet gel was produced. The wet gel was then dried at 120 °C for 12 h under an ambient atmosphere, which yields porous solid matrices called a xerogel. The final form of the yellow phosphor was obtained by annealing the precursor at two different temperatures. Initially, the precursor was annealed at 400 °C for 3 h, and then the annealing temperature was further increased to 800 °C and maintained at this temperature for 5 h; the rate of temperature increase was set to 2 °C /min for these two steps. Likewise, Ca_5_Zn_4(1-x)_In_4x_(VO_4_)_6_ (CZIV) (where x = 0.02, 0.04, and 0.06) phosphors were synthesized using similar experimental conditions as mentioned above for different concentrations of In^3+^ ions, and we determined the optimal In^3+^ ion concentration to be 2 mol% (i.e., x = 0.02) for CZIV phosphors. For the Ca_5_Zn_3.92_In_0.08_(V_(1-y)_Ta_y_O_4_)_6_ (CZIVT) (where y = 0.005, 0.01, 0.03, 0.05 and 0.1) phosphors, the required amount of citric acid was dissolved in 100 ml of DI water in an another beaker, and then tantalum chloride was added to this solution under continuous magnetic stirring because the solubility of tantalum chloride is poor in alkaline solutions because it is water soluble when combined with citrate complexes^35^. The remaining experimental procedure was the same as for the CZV and CZIV phosphors. The experiment was repeated for different concentrations of Ta^5+^ ions. The general characterizations are presented in the supporting information.

### Fabrication of PC-LEDs

A near-UV LED chip with an excitation wavelength at 380 nm was used for the fabrication of the pc-LEDs. The 100% CZIVT phosphor was used for sample 1 pc-LED, and the 60% CZIVT + 40% BAM mixed phosphors were used for sample 2 pc-LED. The phosphor paste was prepared by dispersing the required amounts of phosphor powders in translucent silicone epoxy in a 1:2 weight ratio. To obtain a uniform dispersion, the phosphor paste was kept in a planetary mixer for 10 min. Then, the phosphor paste was kept in a vacuum desiccator to remove air bubbles. Finally, the near-UV LED chip was encapsulated with the phosphor paste and then allowed to harden at 130 °C for 60 min.

## Additional Information

**How to cite this article**: Pavitra, E. *et al*. Novel rare-earth-free yellow Ca_5_Zn_3.92_In_0.08_(V_0.99_Ta_0.01_O_4_)_6_ phosphors for dazzling white light-emitting diodes. *Sci. Rep.* **5**, 10296; doi: 10.1038/srep10296 (2015).

## Supplementary Material

Supplementary Information

## Figures and Tables

**Table 1:**
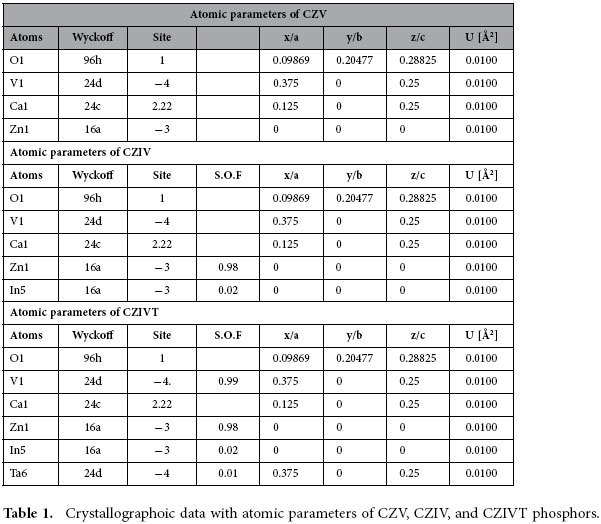
Crystallographoic data with atomic parameters of CZV, CZIV, and CZIVT phosphors.

**Table 2:** Crystallographic phase data and bond lengths of the CZV, CZIV, and CZIVT phosphors.

**Crystallographic data**	**CZV**	**CZIV**	**CZIVT**
Crystal system	Cubic	Cubic	Cubic
Space group	Ia-3d (230)	Ia-3d (230)	Ia-3d (230)
Cell parameters (Å)	a = 12.4668 (0)	a =12.4636 (0)	a =12.4784 (1)
Cell volume (Å)^3^	1937.60 (2)	1936.13 (1)	1943.02 (6)
R-factors (%)	χ^2^ = 5.814	χ^2^ = 6.036	χ^2^ = 5.342
	R_*p*_ = 9.79	R_*p*_ = 10.19	R_*p*_ = 9.41
	R_*wp*_ = 6.04	R_*wp*_ = 6.97	R_*wp*_ = 6.93
Bond lengths(Å)	V-O = 1.732	V-O = 1.732	V-O = 1.734
	Zn-O = 2.026	Zn-O = 2.025	Zn-O = 2.028
	Ca-O = 2.618	Ca-O = 2.618	Ca-O = 2.620
	V-V = 3.817	V-V = 3.816	V-V = 3.820
	V-Zn = 3.485	V-Zn = 3.484	V-Zn = 3.489
	V- Ca = 3.117	V-Ca = 3.116	V-Ca = 3.121

**Table 3:** CCT, CRI, and CIE values of the sample 1, sample 2, and commercial YAG:Ce^3+^ phosphors.

**Sample name**	**CCT (K)**	**CRI**	**CIE (x, y)**
**Sample 1**	4757	70.70	(0.375, 0.464)
at 100 mA			
			
**Sample 2**			
at 100 mA	4950	78.10	(0.333, 0.399)
at 200 mA	5089	79.40	(0.326, 0.388)
at 260 mA	5231	82.51	(0.317, 0.370)
**YAG:Ce**^**3+**^	4555	65	(0.425, 0.454)
**YAG:Ce**^**3+**^** + Blue LED**	7756	75	(0.292, 0.395)
