# Supplementary material for: Novel rare-earth-free yellow Ca5Zn3.92In0.08(V0.99Ta0.01O4)6 phosphors for dazzling white light-emitting diodes
Source: Sci Rep. 2015 May 20;5:10296. doi: 10.1038/srep10296 (PMC4438725; doi:10.1038/srep10296)
Supplement: Supplementary Information [file srep10296-s1.pdf]

SCIENTIFIC REPORTS

Supplementary Information

Novel rare-earth-free yellow $Ca_5Zn_{3.92}In_{0.08}(V_{0.99}Ta_{0.01}O_4)_6$ phosphors for dazzling white light-emitting diodes

E. Pavitra[1†], G. Seeta Rama Raju[1†], Jin Young Park[2], Lili Wang[2], Byng Kee Moon[2] and Jae Su Yu[1]*

[1]Department of Electronics and Radio Engineering, Kyung Hee University, Yongin 446-701, Republic of Korea

[2]Department of Physics, Pukyong National University, Busan 608-737, Republic of Korea

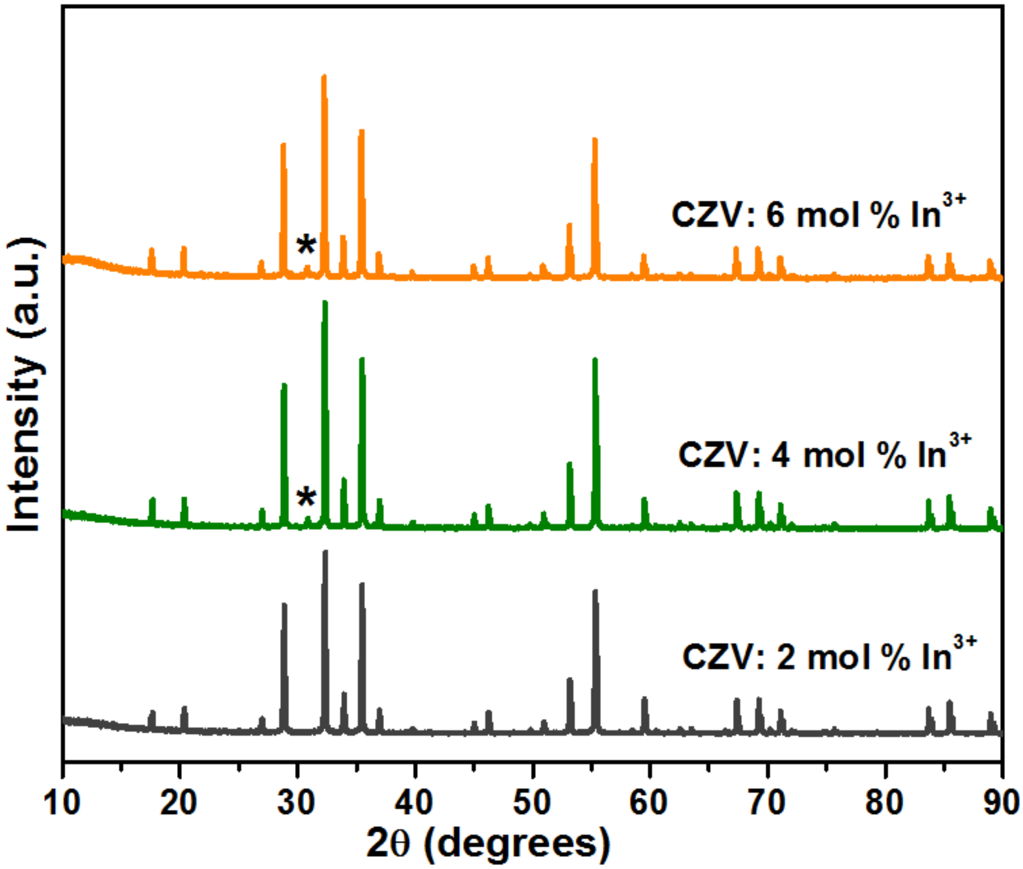

Fig S1│ XRD patterns of CZIV. XRD patterns of the CZIV phosphors as a function of In^{3+} ion concentration

(* mark represents the impurity peak of In_2O_3 (JCPDS No. 71-2194))

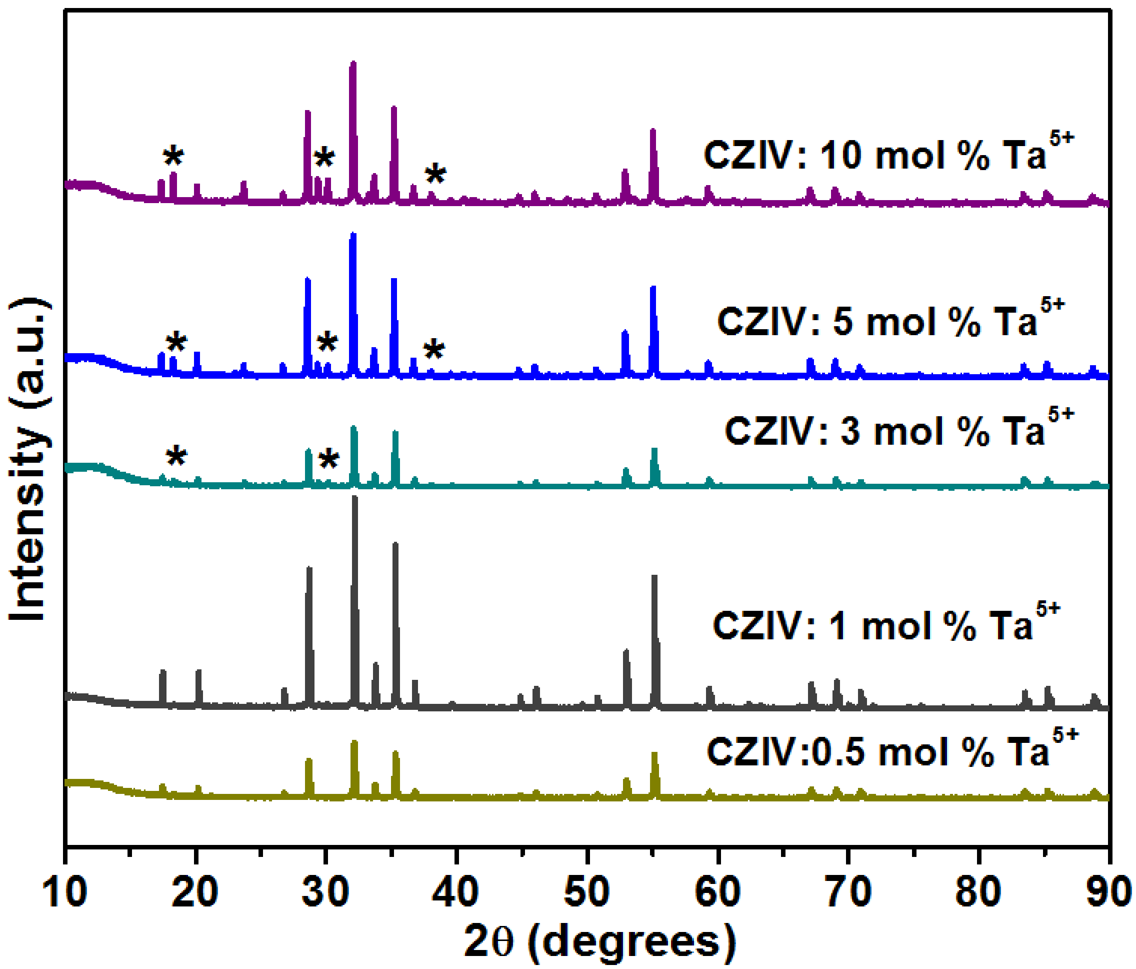

Fig S2│XRD patterns of CZIVT. XRD patterns of the CZIVT phosphors at different concentrations of Ta^{5+} ions

(* indicates the impurity peaks of Ta_2O_5 (JCPDS No. 73-2323))

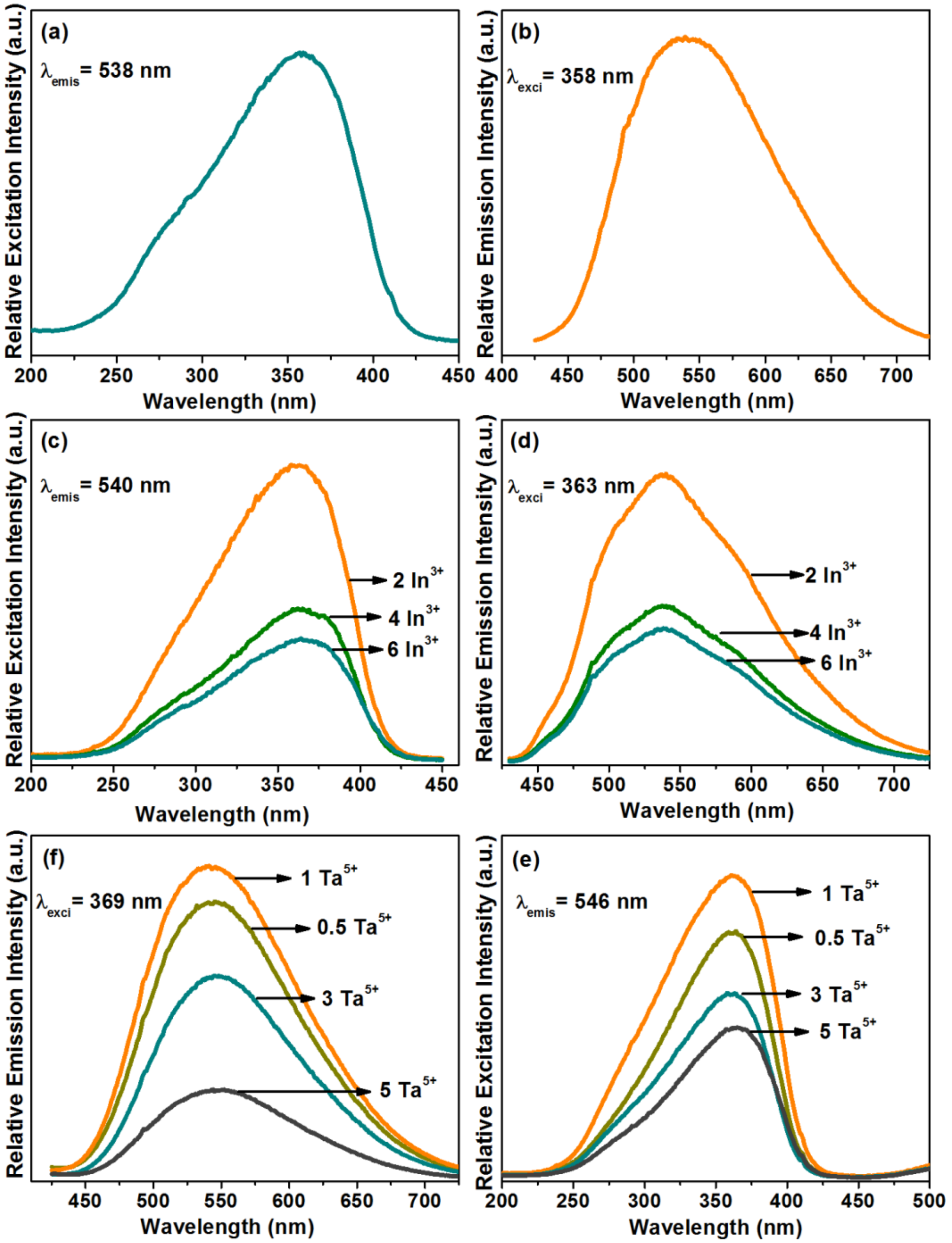

Fig S3│Excitation and emission properties of CZV, CZIV, CZIVT phosphors. (a) PLE and (b) PL emission spectra of the CZV phosphors by monitoring the corresponding excitation and emission wavelengths. (c) PLE and (d) PL emission spectra of the CZIV phosphors as a function of In^{3+} ion concentration. (e) PLE and (f) PL emission spectra of the CZIVT phosphors at different concentrations of Ta^{5+} ions.

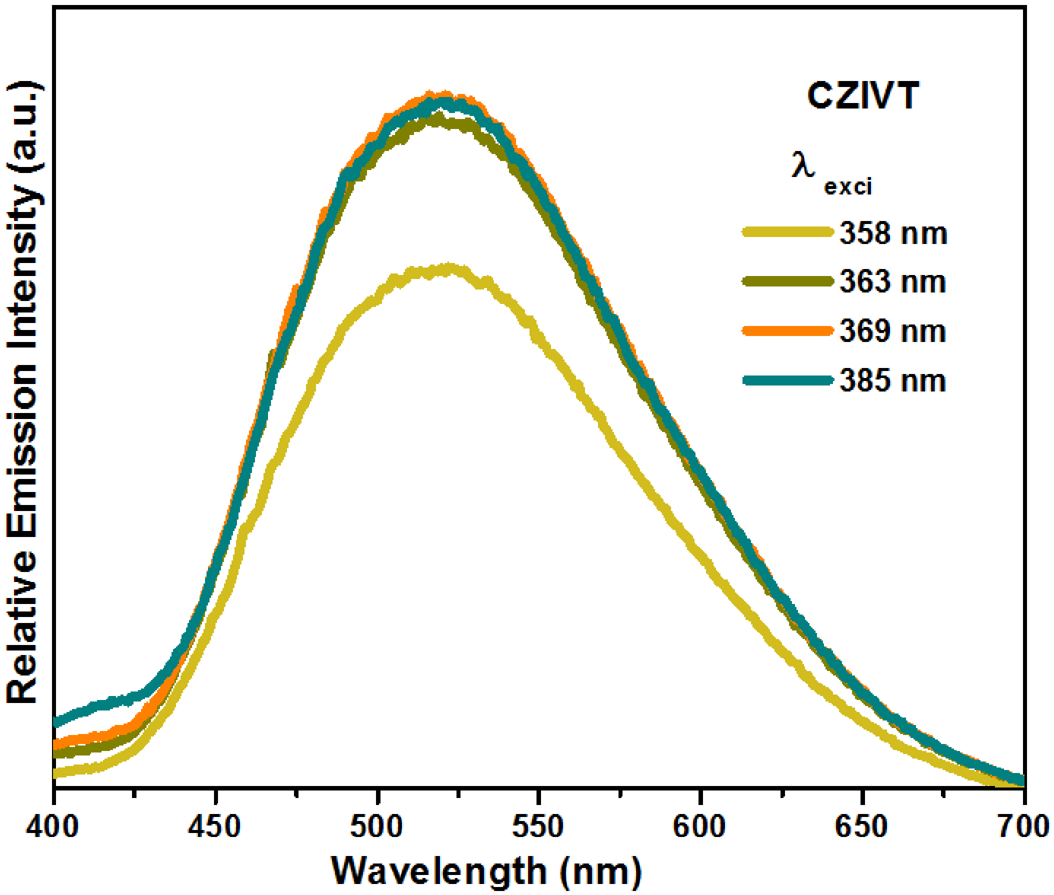

Fig S4│Luminescence properties of CZIVT. Emission spectra of the CZIVT phosphors at different excitation wavelengths.

Characterizations:

The XRD patterns of the samples were verified on Mac Science (M18XHF-SRA) X-ray powder diffractometer with CuKα= 1.5406 Å. The morphology of the samples was examined by field-emission SEM (FE-SEM, JEOL JSM-6700) and field-emission TEM (FE-TEM, JEOL JEM-2100F) images. The room-temperature PL spectra were recorded on a Photon Technology International (PTI, USA) fluorimeter with a Xe-arc lamp of 60 W power and the lifetime was measured with a phosphorimeter attachment to the main system with a Xe-flash lamp (25 watt power).
